# Genetic Susceptibility to Insulin Resistance and Its Association with Estimated Longevity in the Hungarian General and Roma Populations

**DOI:** 10.3390/biomedicines10071703

**Published:** 2022-07-14

**Authors:** Peter Piko, Nardos Abebe Werissa, Roza Adany

**Affiliations:** ELKH-DE Public Health Research Group, Department of Public Health and Epidemiology, Faculty of Medicine, University of Debrecen, 4032 Debrecen, Hungary; piko.peter@med.unideb.hu (P.P.); nardos.abebe@med.unideb.hu (N.A.W.)

**Keywords:** optimized genetic risk score, HOMA—IR, Hungarian, insulin resistance, estimated longevity, Roma, single nucleotide polymorphism, type 2 diabetes

## Abstract

Diabetes mellitus is a major public health problem with a wide range of prevalence among different ethnic groups. Early recognition of pre-diabetes is important to prevent the development of the disease, its complications, co-morbidities, and consequently early death. Insulin resistance (IR) is considered a condition that precedes type 2 diabetes; thus, understanding its underlying causes (genetic and non-genetic factors) will bring us closer to preventing it. The present study aimed to investigate the genetic susceptibility to IR and its impact on estimated longevity in populations with different ethnic origins using randomly selected samples of 372 Hungarian general (HG, as a reference with Caucasian origin) and 334 Roma participants (largest ethnic minority in Europe, with a northern India origin). In the present study, we used the Homeostasis Model Assessment—Insulin Resistance (HOMA—IR) to identify people with IR (>3.63) at the population level. To investigate the genetic predisposition to IR, 29 single nucleotide polymorphisms (SNPs) identified in a systematic literature search were selected and genotyped in sample populations. In the analyses, the adjusted *p* < 0.0033 was considered significant. Of these 29 SNPs, the commutative effects of 15 SNPs showing the strongest association with HOMA—IR were used to calculate an optimized genetic risk score (oGRS). The oGRS was found nominally significantly (*p* = 0.019) higher in the Roma population compared to HG one, and it was more strongly correlated with HOMA—IR. Therefore, it can be considered as a stronger predictor of the presence of IR among the Roma (AUCRoma = 0.673 vs. AUCHG = 0.528). Furthermore, oGRS also showed a significant correlation with reduced estimated longevity in the Roma population (β = −0.724, 95% CI: −1.230–−0.218; *p* = 0.005), but not in the HG one (β = 0.065, 95% CI: −0.388–0.518; *p* = 0.779). Overall, IR shows a strong correlation with a genetic predisposition among Roma, but not in the HG population. Furthermore, the increased genetic risk of Roma is associated with shorter estimated longevity, whereas this association is not observed in the HG one. Increased genetic susceptibility of Roma to IR should be considered in preventive programs targeting the development of type 2 diabetes, which may also reduce the risk of preventable premature death among them.

## 1. Introduction

Epidemiological studies warn of an increase in diabetes prevalence worldwide [1]. Diabetes, besides affecting many, was responsible for approximately 12.2% of all deaths globally in 2021 [1]. Currently, it is listed in the top 10 causes of death worldwide [2,3], and together with cardiovascular diseases (CVDs), cancer, and respiratory diseases, it accounts for more than 80% of premature deaths caused by non-communicable diseases (NCDs) globally [4]. Diabetes places a huge burden on the overall health status of an individual and the health system and economy of a country. In the last fifteen years, the health expenditure for diabetes has grown by 316% [1]. Epidemiological studies emphasize that the prevention of a disease is the most (cost) effective way to reduce its burden [5].

The distribution of people with diabetes is uneven across the world and disproportionately affects specific population groups. Eighty percent of deaths directly caused by diabetes occur in low- and middle-income countries [6]. In Europe, the age-adjusted comparative prevalence of type 2 diabetes mellitus (T2DM) varied between 3% (Ireland) and 14.5% (Turkey) in 2021 [1]. In high-income countries, there are significant disparities between the general population and racial/ethnic minorities and those of low socioeconomic status [7]. An analysis of data from more than 400,000 people in the United Kingdom showed that the prevalence of T2DM was higher in Asian (7.69%, 95% CI 7.46 to 7.92) and black (5.58%, 95% CI 5.35 to 5.81) ethnic groups than in white group (5.04%, 95% CI 4.95 to 5.13) [8].

Insulin resistance (IR), which could be defined as an impaired ability of insulin to stimulate glucose disposal or the inability of cells to utilize glucose at normal insulin concentrations according to their specific functions, is considered a condition that can precede T2DM (which is the most common form of diabetes) [9,10]. In addition to its significant role in the development of T2DM, IR is also accepted as the central risk factor in the development of metabolic syndrome (MetS) and also fuels the development of other cardiometabolic risk factors such as dyslipidemia, abdominal obesity, hypertension, as well as CVDs in general [11,12]. Understanding the processes underlying the pathways leading to IR can help to develop preventive interventions that effectively target these diseases and thereby influence the life expectancy of individuals [13,14,15].

The development of IR is strongly influenced by environmental/lifestyle factors [16,17,18] such as physical (in)activity [19,20,21,22], age [23,24], obesity [25,26], smoking [27,28], and low-fiber diet [29], and it is generally accepted that individual risk is also strongly influenced by inheritable genetic factors [30,31], which may partly explain the differences in diabetes prevalence between ethnic groups [32]. The currently available literature suggests that genetic factors account for 25% to 40% of a person’s vulnerability to IR [31]. Genome-wide association studies (GWAS) unequivocally support the involvement of genetic factors (such as single nucleotide polymorphisms, SNPs) in the development of IR [33,34]. These identified gene variants individually account for only a small fraction to explain the disease; therefore, aggregating various SNPs together through polygenic, also known as genetic, risk score (GRS) can strongly increase their informative and predictive power [33,35].

In multinational and multicultural regions with multiethnic communities such as Europe, an approach that considers both conventional (such as age, sex, obesity, etc.) and genetic factors is becoming increasingly important to implement both personalized public health programs and integrated community-based interventions to effectively prevent multifactorial diseases.

With an estimated 10–12 million people, the Roma population is the largest minority in Europe, living mainly in the countries of Central and Eastern Europe [36]. The Roma (Gypsies) originated in northern India as a nomadic people and arrived in Europe between the eighth and tenth centuries AD [37]. The general health status of Roma is poor, with a high prevalence of risk factors associated with the development of communicable and NCDs, which partly explains why their life expectancy is assumed to be lower than that of the majority population [38,39,40,41]. The underlying causes are manifold, and their poor socio-economic status may play a prominent role, but genetic predisposition cannot be excluded [42,43,44].

According to the results of our complex health survey carried out on samples of adults aged 20–64 in 2018 [45], the prevalence of IR (based on the Homeostasis Model Assessment—Insulin Resistance (HOMA—IR)) is high in both the Hungarian general (HG) and Roma populations (47.61% and 47.78%, respectively). In addition, it is known from our previous study that the high prevalence of elevated fasting blood glucose or known T2DM among Roma is not due to a high genetic risk (estimated based on SNPs associated with elevated fasting blood glucose levels) but is presumably due to Roma ethnicity and other associated factors [46]. The fasting blood glucose level and the development of T2DM in an individual is largely determined by the individual’s ability to produce insulin and the cellular response to it. However, these characteristics vary not only at the individual but also at the population level worldwide. It is known that the Roma population is of Asian origin and that the association between insulin sensitivity and insulin response in populations of Asian origin is drastically different from that in populations of Caucasian origin [32]. Thus, the question arises: Is the high prevalence of T2DM among Roma due to environmental and lifestyle characteristics and/or to IR-related genetic characteristics?

The main objective of the current study is therefore: (1) to explore whether IR in the Hungarian general (with Caucasian origin) and Roma (with Indian origin) populations have a genetic background; (2) to compare the genetic risk load between the two study populations; (3) to evaluate the predictive ability of genetic factors (using GRS) for IR in the two populations. The findings from this study may increase our knowledge of the genetic susceptibility of the Hungarian general and Roma population to IR. Furthermore, it can be used for the identification of groups at increased risk that could be a way to guide better intervention and prevention strategies for T2DM and CVDs. In addition, the relationship between genetic susceptibility to IR and longevity is also estimated.

## 2. Materials and Methods

### 2.1. Sample Populations

The database used for the present study is based on a cross-sectional three-pillar survey (physical, laboratory examinations, and questionnaire-based) carried out in 2018. Information on data collection and sampling is detailed elsewhere [45]. In brief, both the Roma and Hungarian general samples were collected from 2 counties in northeastern Hungary (Szabolcs-Szatmár-Bereg and Hajdú-Bihar), where the majority of segregated Roma settlements are found in Hungary and where the Roma representation is high. First, a randomized sample selection process was carried out, whereby 25 Roma colonies with more than 100 inhabitants were selected. For each colony, 20 households were selected, and for each household, one person aged between 20 and 64 was interviewed. These interviews were conducted in the respondent’s households by Roma university students under the supervision of public health coordinators. Participant ethnicity was assessed by self-report. The HG population was collected from general practitioners (GP) registered people living in private households in Szabolcs-Szatmár-Bereg and Hajdú-Bihar counties. From 20 randomly selected GP practices, 25 randomly selected individuals aged between 20 and 64 years were selected to participate in the survey.

**Figure 1:**
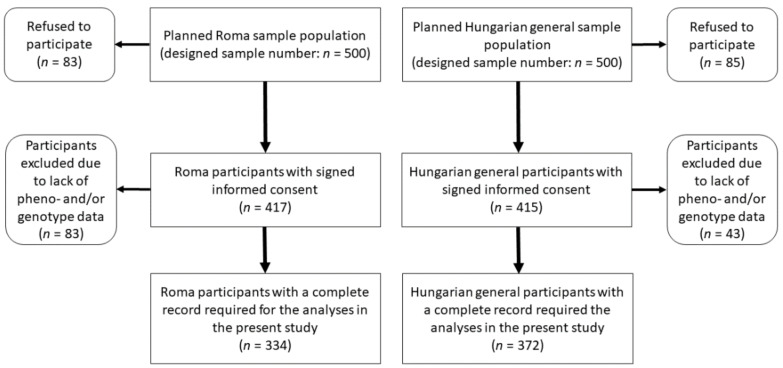
Flowchart showing the process of sample selection for Roma and Hungarian general populations.

The planned sample size for both study groups was 500 and 500 participants, but the final study sample, which included participants only with complete records, consisted of 706 participants, of whom 372 were from the HG and 334 from the Roma population (see more details in Figure 1). Anthropometric, demographic, socioeconomic, and health-related data survey participants were recorded, and fasting blood samples (native and EDTA-anticoagulated) were also collected for laboratory testing. Findings obtained in the survey were registered in a database previously described in detail [45].

### 2.2. Data Used to Identify Insulin Resistance in Sample Populations

The prevalence of IR largely varies from country to country, ranging from 17% to 51% [47,48], but the comparability of these data is questionable. One of the limitations of estimating the prevalence of IR at the population level is the variety of methods/indices used to define it. The hyperinsulinemic-euglycemic clamp technique is considered the most accurate technique and the gold standard for IR measurement [49], but it is almost impossible to use this invasive method in population-level studies [50,51]. Several simple and non-invasive methods have been identified and are available as surrogate markers for the assessment of IR, of which the HOMA—IR index is the most widely used and convenient way to quantify IR at the population level [51,52,53].

For the present study, the following data were used: ethnicity, age, sex, body mass index (BMI), antihypertension treatment, lipid-lowering treatment, antidiabetic treatment, as well as fasting insulin and blood glucose values to calculate HOMA—IR. Fasting blood glucose was considered elevated if it reached or exceeded 7 mmol/L, while fasting insulin was considered elevated if it was greater than 20 mU/L. Finally, individuals with a HOMA—IR value greater than 3.63 [51] were considered to be IR with a high chance for developing diabetes [54].

### 2.3. DNA Extraction, SNP Selection, Genotyping, Testing Hardy-Weinberg Equilibrium, and Linkage Disequilibrium

The MagNA Pure LC system (Roche Diagnostics, Basel, Switzerland) was used to extract DNA from EDTA-anticoagulated blood samples using the DNA Isolation Kit-Large volume method. The extraction was performed according to the manufacturer’s instructions.

Using online search engines such as PubMed, Ensemble, and HuGE navigator, a systematic literature search was conducted to identify SNPs statistically significantly associated with IR and/or T2DM. SNPs were selected based on the consistency of associations in European and non-European populations with statistically acceptable sample sizes.

The literature search identified a total of 29 SNPs (10 SNPs associated with IR and 19 SNPs associated with T2DM), and these were genotyped using the MassARRAY platform (Sequenom Inc., San Diego, CA, USA) with iPLEX Gold chemistry in the Mutation Analysis Core Facility (MAF) of the Karolinska University Hospital, Sweden. Validation, concordance analysis, and quality control were conducted by the MAF, according to their protocols. Hardy-Weinberg equilibrium and linkage disequilibrium (LD) structure of the genotyped SNPs were calculated by Haploview software version 4.2 (Broad Institute, Cambridge, MA, USA).

### 2.4. Identification and Coding of the Genetic Model Best Associated with HOMA—IR for SNPs

For each SNP, we examined which of the three widely used genetic inheritance models (i.e., codominant, dominant, and recessive) showed the strongest association with HOMA—IR as a continuous outcome. Linear regression analysis (adjusted for ethnicity, age, sex, BMI, antihypertension treatment, lipid-lowering treatment, and antidiabetic treatment) was carried out for testing the correlation of each SNP with HOMA—IR as a continuous outcome. R-squared (the higher the better) and *p* values (the lower the better) were considered in the selection process for identifying the most fitted heritability model [55]. For each SNP used in the calculation of the optimized genetic risk score (oGRS), the most highly correlated heritability model with HOMA—IR level was considered.

Code was given for each SNP based on the criteria of the genetic model of inheritance. Hence, for the:

Codominant genetic model: homozygote genotype with risk allele was labeled as 2, whereas the heterozygote gene as 1 and 0 was coded for no risk allele.

Dominant genetic model: 2 was coded for the presence of one or two risk alleles, and 0 was coded for the absence of a risk allele.

Recessive genetic model: 2 was counted for the presence of two risk alleles, while 0 was counted for the homozygote gene with the absence of a risk allele and for the heterozygote gene.

### 2.5. Calculation and Optimization of the Genetic Risk Score

The oGRS was calculated using Equation (1), where Gi is the risk score according to the chosen heritability model (see the previous subsection for details).

(1) $$GRS=\sum_{i=1}^{I}Gi$$

The genetic risk model optimization procedure aimed to select SNPs (identified in the systematic literature search) that had the strongest association with HOMA—IR (as an outcome variable) in both study populations. For GRS optimization, adjusted linear regression analyses (for ethnicity, age, sex, BMI, antihypertensive treatment, lipid-lowering treatment, and antidiabetic treatment) were used, and these analyses were performed on a combination of the two populations.

The SNPs were tested in ascending order of *p* value, in which the process consisted of each SNP being inserted into the statistical model one by one, starting from the SNP with the strongest association (with the lowest *p* value), and the association between oGRS and HOMA—IR was examined after each insertion.

SNPs were selected and used for final optimized GRS only if they increased the strength of association of oGRS with HOMA—IR (i.e., decreased *p* value and increased R-squared value). SNPs that did not affect or weakened the association (i.e., increased the *p* value and decreased the R-squared value), were excluded from further analyses.

Based on the oGRS, individuals were classified into three risk groups. The distribution of the oGRS for the combined population (i.e., Hungarian general and Roma populations together) was considered in the formation of the groups. Approximately, the individuals in the lowest 25% were assigned to the low-risk group, the individuals in the middle 50% were assigned to the medium-risk group, and the individuals in the top 25% were assigned to the high-genetic-risk group.

### 2.6. Statistical Analysis

χ^2^ test was used to examine the Hardy-Weinberg equilibrium (HWE) of genotyped SNPs. In addition, it was used to compare the differences between nonquantitative variables amongst the study populations. The Shapiro-Wilk test was used to examine whether the quantitative variables are normally distributed, and if it was necessary, Templeton’s two-step method was considered to transform the non-normal variables into normal ones [56]. Mann-Whitney U test was used to assess the distribution of age, BMI, fasting glucose, fasting insulin, HOMA—IR, and oGRS between the study populations. Multiple linear and logistic regression analyses were used to determine the association between individual SNPs and the aggregate of them (oGRS) and HOMA—IR level (as a continuous variable) and IR status (as a binary variable). Cox regression analysis was used to examine the association of oGRS with age at the onset of IR and estimated longevity separately for study populations. In these analyses, the age of the individual at the time the questionnaire was collected was used as the outcome variable.

All kinds of regression analyses were carried out under the adjusted (age, sex, BMI, antihypertension treatment, lipid-lowering treatment, and antidiabetic treatment) model. When the two populations were examined together, ethnicity was used as a covariate. The seemingly unrelated regression model was used to compare the effect of oGRS measured separately in the two populations. A statistically significant trend between the proportion of individuals with elevated HOMA—IR and oGRS risk categories was tested using the Jonckheere-Terpstra test. Receiver operating characteristic (ROC) analysis was employed to evaluate the discriminatory ability of the oGRS, and the area under the curve (AUC) was used as a diagnostic index. Statistical tests were carried out using IBM SPSS version 26 statistics for Windows (Armonk, NY, USA). When multiple statistical tests were performed simultaneously (all calculations involving the oGRS), the Bonferroni correction was applied (conventional *p* value of 0.05 divided by the number of independent SNPs).

### 2.7. Ethical Statement

All subjects gave their informed consent to participate in the study. The study was conducted following the ethical standards of the institutional and national research committees, in harmony with the declaration of Helsinki. The study was conducted in accordance with the Declaration of Helsinki, and the protocol was approved by the Ethics Committee of the Hungarian Scientific Council on Health (61327-2017/EKU).

## 3. Results

### 3.1. Characteristics of the Study Populations and Results of the HWE and LD Analyses

Samples with incomplete geno- and/or phenotype data were excluded from further analyses; thus, a total of 706 individuals (HG: *n* = 372 and Roma: *n* = 334) were involved in the study. The only significant difference in the main characteristics considered in the present study of the two populations was the sex distribution (Table 1).

Regarding genotype distribution, no significant differences from HWE were found in either population. The results of LD can be seen in more detail in Supplementary Figure S1.

### 3.2. Results Obtained from the Analysis of the Determination of the Best Fitting Genetic Model for SNPs

Multiple linear regression analyses (adjusted for ethnicity, age, sex, BMI, antihypertension treatment, lipid-lowering treatment, and antidiabetic treatment) were carried out to assess the correlation of SNPs with HOMA—IR (as a continuous outcome). The three most used genetic models of inheritance (namely codominance, recessive, and dominance) were tested for each SNP to determine which one shows the strongest association with HOMA—IR. During the determination process, models with higher *p* value were excluded, and the model with the lowest *p* value was chosen. Eleven SNPs showed the strongest association with HOMA—IR for the recessive, seven for the codominant, and eleven for the dominant inheritance model. For more detailed results of the calculation see, Table 2.

### 3.3. Results of the Calculation and Optimization of the GRS Model and Determination of the Bonferroni Corrected p Value

In calculating the oGRS, we sought to select SNPs that, based on linear regression analysis, strengthen the association between GRS and HOMA—IR. Starting with the SNP with the strongest association (rs7961581; β = 1.042, 95% CI: 0.406–1.679, *p* = 0.001), we added each SNP one by one into the statistical model (adjusted for age, sex, BMI, antihypertensive, lipid-lowering and antidiabetic treatment). All the SNPs that enhanced the association of the model (decreased *p* value and increased R^2^) were maintained and utilized for the calculation of oGRS. Conversely, those SNPs that weakened the association of the model (increased *p* value and decreased R^2^) were omitted. During the optimization process, 15 SNPs (and the same number of genes, for more details, see Supplementary Table S1) were selected.

Based on the 15 SNPs identified in the optimization, the Bonferroni corrected *p* value was set at 0.0033 and was considered significant in all analyses where oGRS was included.

### 3.4. Comparison of the Distribution of oGRS Value and Its Association with Fasting Insulin, Fasting Glucose Levels, and HOMA—IR in the Study Populations

**Figure 2:**
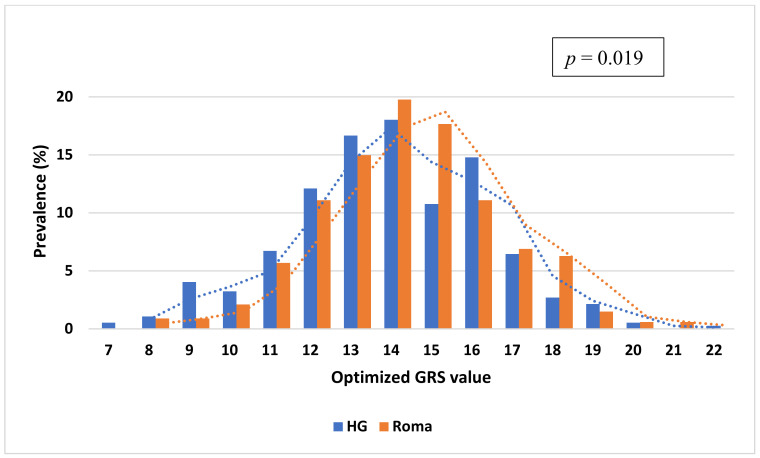
Distribution of genetic risk score optimized for HOMA—IR based on fifteen single nucleotide polymorphisms (selected in the GRS optimization process) in the Roma and Hungarian general (HG) populations.

The mean value of oGRS was 13.82 with a range of 7.0–22.0 in the HG population and 14.28 with a range of 8.0–21.0 among the Roma. The distribution of the oGRS between the study populations showed a significant difference (*p* = 0.019), and the Roma population carried higher oGRS compared to the HG one (see more details in Figure 2).

Multiple linear regression analysis was carried out to examine the association between fasting insulin and glucose levels and oGRS after adjusting for age, sex, BMI, antihypertensive, lipid-lowering, and antidiabetic treatment. The oGRS was significantly associated with fasting insulin levels as a continuous outcome in both the HG (β = 1.151, 95% CI: 0.485–1.818; *p* < 0.001) and Roma (β = 2.171, 95% CI: 1.372–2.971; *p* < 0.001) populations. If insulin level was considered as a binary outcome (i.e., elevated or normal) the oGRS showed a significant association with elevated insulin level only in the Roma population (odds ratio (OR) = 1.246 95% CI: 1.095–1.418; *p* < 0.001), but not in the HG one (OR = 1.065, 95% CI: 0.953–1.083; *p* = 0.266). Nominal significant associations of oGRS with fasting glucose level (β = 0.090, 95% CI: 0.013–0.168; *p* = 0.022) and elevated fasting glucose status (OR = 1.284, 95% CI: 1.044–1.579; *p* = 0.018) could be detected in the Roma population, but no association for either level (β = 0.031, 95% CI: −0.035–0.097; *p* = 0.357) or status (OR = 1.078, 0.901–1.289; *p* = 0.413) could be seen in the HG population.

The effect of oGRS on fasting insulin (*p* = 0.053) and blood glucose (*p* = 0.264) levels showed no significant difference when comparing the two populations.

The oGRS showed a significant association with HOMA—IR as a continuous (β = 0.701, 95% CI: 0.434–0.969; *p* < 0.001) and binary outcome (OR = 1.297, 95% CI: 1.142–1.474; *p* < 0.001) in the Roma population, while in the HG population, oGRS showed a significant association with HOMA—IR only as a continuous outcome variable (β = 0.373, 95% CI: 0.156–0.590; *p* < 0.001), and not as a categorical one (OR = 1.059, 0.958–1.171; *p* = 0.259).

**Figure 3:**
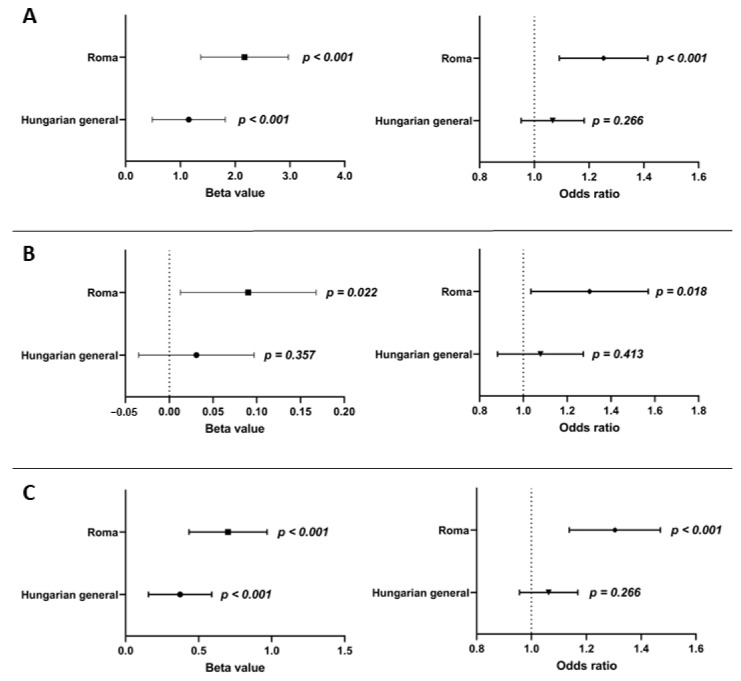
Effect of oGRS on the fasting insulin level (**A**), fasting glucose level (**B**), and HOMA—IR (**C**) as a continuous (in β value) and binary (in OR) outcome in the Roma and Hungarian general populations. The association was evaluated by using an adjusted (for age, sex, BMI, and treatments) linear regression model. Bonferroni corrected *p* value: 0.0033.

The magnitude of the effect of oGRS on HOMA—IR was compared between the study populations, and it significantly (*p* < 0.001) increased the risk of IR in the Roma population to a greater extent than in the HG one. See more details in Figure 3.

### 3.5. The Effect of Genetic (oGRS) and Conventional (Age, Gender, BMI, and Treatments) Risk Factors and the Combination of Them on Insulin Resistance in the Study Populations

The discriminatory power of genetic (oGRS), and the conventional (age, sex, BMI, and treatments) and combination of these (genetic and conventional) factors on IR in both study populations were examined. The oGRS has an AUC of 0.528 and 0.673 in the HG and Roma populations, respectively. The AUC of conventional risk factors was 0.722 and 0.756 for the HG and Roma populations, respectively. Using genetic and conventional risk factors together, the AUC was 0.723 and 0.785 for the HG and Roma populations, respectively.

**Figure 4:**
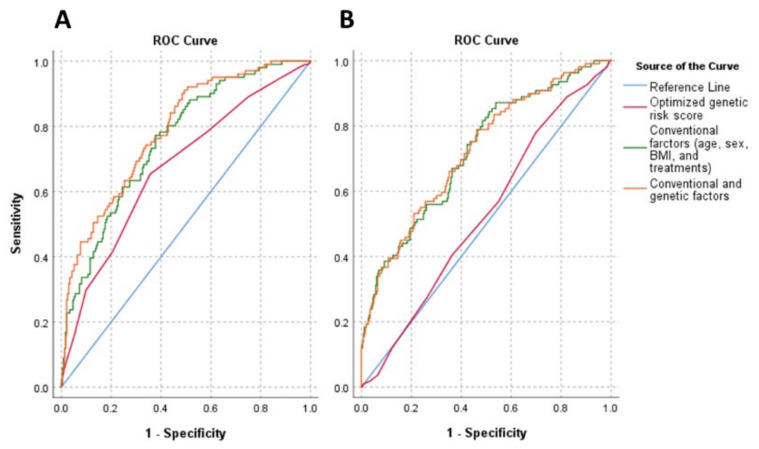
Receiver operating characteristic (ROC) curves and AUC for discriminating insulin resistance (HOMA—IR > 3.63). Graphs show ROC curves and AUC for conventional risk factors (age, sex, BMI, and treatments) by the green line, optimized genetic risk score by the red line, and combinations of these by the orange line for the Roma (**A**) and Hungarian general (**B**) populations. The reference was marked by a light blue line, indicating zero predictive value of the model. Conventional risk factors: AUC_Roma_: 0.756, AUC_HG_: 0.722; optimized genetic risk score: AUC_Roma_: 0.673, AUC_HG_: 0.528; combinations of conventional risk factors and optimized genetic risk score: AUC_Roma_: 0.785, AUC_HG_: 0.723.

The discriminatory power of conventional risk factors was significantly (*p* < 0.001) stronger in the HG population (AUC_conv._ = 0.722 vs. AUC_oGRS_ = 0.528; *p* < 0.001) compared to the genetic one, while it was not in the Roma population (AUC_conv._ = 0.756 vs. AUC_oGRS_ = 0.673; *p* = 0.048). Compared to the conventional risk factors, the combined one did not show a significant improvement in discriminatory ability in either population (HG: AUC_conv._ = 0.722 vs. AUC_com._ = 0.723, *p* = 0.795; Roma: AUC_conv._ = 0.756 vs. AUC_com_ = 0.785, *p* = 0.069). For both populations, the combined model resulted in a significantly stronger discrimination index than the oGRS alone (HG: AUC_oGRS._ = 0.528 vs. AUC_com._ = 0.723, *p* < 0.001; Roma: AUC_oGRS_ = 0.673 vs. AUC_com_ = 0.785, *p* < 0.001). For more details, see Figure 4.

### 3.6. Association of Genetic Risk Groups with Insulin Resistance in the Study Populations

The low-risk group consisted of people with an oGRS value that was 12 and below (prevalence in the combined population: 24.4%), the medium-risk group consisted of people with oGRS of 13–15 (prevalence in the combined population: 48.7%), and the high-risk group consisted of people with oGRS 16 and above (prevalence in the combined population: 26.9%). In the Roma population, a significant (*p* < 0.001) positive trend was observed between the genetic risk group categories and the proportion of IR individuals (low: 15.9%, medium: 27.4%, and high: 46.7%). This was not observed in the HG population (low: 23.3%, medium: 32.5%, and high: 31.0%; *p* = 0.224). See more details in Table 3.

Furthermore, adjusted multinomial logistic regression was used to examine how genetic risk groups affect the risk of developing IR in the study populations. In the analyses, the low-risk group was used as a reference. In the Roma population, belonging to the medium (OR = 2.532, 95% CI: 1.398–4.588; *p* = 0.002) or the high risk (OR = 4.261, 95% CI: 1.841–9.862; *p* < 0.001) groups significantly increased the risk for the development of IR. In the HG population, neither belonging to the medium (OR = 0.902, 95% CI: 0.499–1.629; *p* = 0.732) nor the high risk (OR = 1.696, 95% CI: 0.847–3.396; *p* = 0.136) groups increased the risk of IR. See more details in Supplementary Figure S2.

### 3.7. Association of oGRS with Early Onset of Insulin Resistance and Estimated Longevity

Cox regression was used to examine the association of oGRS with the estimated time of onset of IR (age of individuals with a HOMA—IR greater than 3.63 at the time of examination) in the study populations. For the Roma population, there is a strong significant association between the time of onset of IR and estimated genetic risk by oGRS (Hazard Ratio (HR) = 1.217, 95% CI: 1.117–1.327, *p* < 0.001). A similar positive correlation was also shown in the HG population, but it was not statistically significant (HR = 1.048, 95% CI: 0.970–1.132; *p* = 0.232).

**Figure 5:**
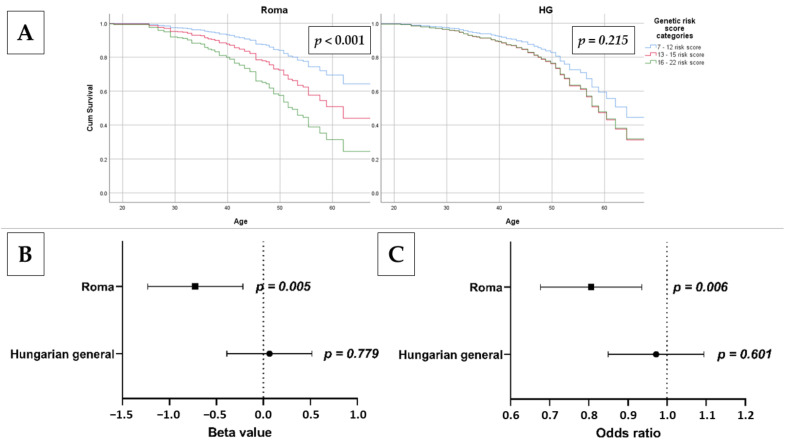
The association of oGRS with age at onset of IR using Cox regression analysis (**A**) and estimated longevity as continuous (**B**) and categorical: 20–34-year-old group was used as reference vs. 50–64 years old; (**C**) outcome in the Roma and Hungarian general (HG) populations. Bonferroni corrected *p* value: 0.0033.

The effect of risk categories based on oGRS on the time of onset of IR was investigated. The low-risk category was used as a reference. For the Roma population, compared to the low-risk group, an increased risk was present for those in the medium-risk (HR = 1.856, 95% CI: 0.955–3.609, *p* = 0.068) and high-risk (HR = 3.182, 95% CI: 1.620–6.250, *p* = 0.001) groups. For the HG population (similar to the continuous variable oGRS), belonging to the medium- and high-risk groups showed a non-significant positive correlation with early onset of IR, but no significant difference in risk between the two groups was detected (HR_medium_ = 1.438, 95% CI: 0.881–2.348; *p* = 0.147 and HR_high_ = 1.415, 95% CI: 0.817–2.452; *p* = 0.216). See more details in Figure 5.

Adjusted linear regression was used to analyze the association of oGRS with age as a continuous and categorical outcome (20–34 years old as reference vs. 50–64 years old). In the Roma population, there was a significant association between age as a continuous (β = −0.724, 95% CI: −1.230–−0.218; *p* = 0.005) and as a categorical (OR = 0.799, 95% CI: 0.680–0.939; *p* = 0.006) outcome variable. In the HG population, there is no significant association between age as a continuous (β = 0.065, 95% CI: −0.388–0.518; *p* = 0.779) or categorical (OR = 0.967, 95% CI: 0.852–1.097; *p* = 0.601) outcome variable and oGRS. See more details in Figure 5.

## 4. Discussion

It is generally accepted that IR and the attendant compensatory hyperinsulinemia are causally related to MetS, a cluster of symptoms based on interconnecting disturbances in carbohydrate and lipid metabolisms resulting in abdominal obesity, glucose intolerance, dyslipidemia, high blood pressure, and vascular dysfunction [57]. The prevalence of MetS is rapidly increasing with the spread of the Western lifestyle across the world, and now it is considered a global health problem [58], although its prevalence varies in a wide range among different ethnic groups [59,60]. Based on the results of two health examination surveys performed on the HG population aged 20–64 years in 2006 and 2018, we could show that the prevalence of MetS increased significantly in the period examined (from 34.9% to 42.2%, *p* = 0.035) due to the increased prevalence of raised blood pressure (from 45.6% to 57.0%, *p* = 0.002) and raised fasting glucose concentration (13.2% vs. 24.8%, *p* < 0.001). The increase mainly affected the younger (20–34 years old) age group (12.1% in 2006 vs. 31.6% in 2018, *p* = 0.001) [61]. The risk for MetS increased significantly in the 20–34 (OR = 1.10, *p* = 0.038) and 35–49 (OR = 1.07, *p* = 0.048) year age groups of Roma in a much shorter period of time, i.e., in the 2018 study population compared to the 2011 one [62]. Nevertheless, the number of published IR studies in the HG population [45] is limited and sporadic in the Roma population [45,63]. Furthermore, the genetic determination of IR in these populations has not been previously investigated. In the absence of this knowledge, there are difficulties in implementing effective interventions (either tailored to the individual or population/ethnicity).

Our present study was designed to fill this knowledge gap. We examined the individual and cumulative effects of twenty-nine SNPs (associated with T2DM and/or IR) on HOMA—IR (one of the best indicators of IR). In the individual effect analyses, we identified five SNPs (rs7961581, rs1801282, rs6822892, rs13266634, and rs4430796) that showed at least nominal significant association with HOMA—IR. Subsequently, the GRS optimization process resulted in the identification of 15 SNPs that were used to model oGRS. The value of oGRS was found to be nominally significantly (*p* = 0.019) higher in the Roma population, indicating an elevated genetic risk. The oGRS showed a stronger correlation of IR in the Roma population compared to HG one, regardless of whether the HOMA—IR examined either as a continuous or a categorical variable and fasting blood glucose and insulin levels as a continuous variable.

A small number of studies explored and reported the association between GRS and HOMA—IR and/or IR. These studies reinforce our findings that genetic factors impact the development of IR. Lotta et al. built a GRS comprising fifty-three SNPs and investigated its association with IR in adults [33]. Similarly, Graae and his colleagues also constructed the same GRS on Danish children and adolescents and detected a significant association with IR [64]. Furthermore, Stančáková et al. also created GRS consisting of nine SNPs that are related to IR trait and demonstrated a significant association with HOMA—IR in adult Finnish men (aged between 45 and 73 years) [35].

For both populations, we examined whether oGRS was associated with age (as a continuous or categorical variable) and found that in the Roma population, there was a significant negative association, whereas in the HG population, there was no significant association. These results suggest that while in the Roma population genetic determinants of IR are associated with longevity, this relationship was not observed in the HG population.

Through various biological mechanisms, IR plays a key role in shortening life expectancy [65], meaning that individuals with elevated HOMA—IR are expected to die at a younger age due to the associated diseases. One possible mechanism is the link between insulin resistance and inflammatory processes. In addition to indicating the presence of IR [66], inflammatory cytokines may have a role in vascular damage [67] and have been associated with obesity [68], T2DM [69], MetS [70], and early mortality [71].

Kanaya and colleagues found that South Asian individuals had a significantly higher degree of IR compared to African American, Hispanic White, and non-Hispanic White individuals when adjusted for age, sex, BMI, waist circumference, smoking, and alcohol use [72]. The Roma population is of (South) Asian origin, having encountered many populations during their migrations, but due to their inbred lifestyle, they have partly preserved the genetic characteristics of the source population [37]. Furthermore, the life expectancy of the Roma population is lower than that of the majority population, irrespective of the host country, which is partly explained by their lifestyle and environmental characteristics [73]. The South Asian origin of the Roma and the higher prevalence of IR among people of South Asian origin (regardless of geographic location) also suggest that IR is genetically determined, and this is associated with the generally lower life expectancy among them based on our results.

It is undeniable that this study has some limitations. For instance, the representation of females in the Roma population was much higher compared to males. The other limitation is that individuals who are above 65 years of age were not included in the study. Accurate ethnic identification is a common challenge in studies such as ours. Roma ethnicity was self-reported, and Roma samples were collected from northeast Hungary, where these individuals are accumulated in segregated colonies. Therefore, this sample cannot be interpreted as a representative sample for the whole Hungarian Roma population. Owing to a lack of information on gene-gene and gene-environment interactions, epigenetic factors, and structural variants, we did not consider them in our analysis. In the current study, 29 SNPs that play a role in the development of IR were considered for the calculation of oGRS. Incorporating a larger number of SNPs may further improve the predictive ability of the GRS model. Nonetheless, adding many SNPs into the GRS model does not necessarily lead to a better predictive ability [74,75]. The present analyses were adjusted for relevant covariates; however, several environmental and lifestyle factors (such as physical inactivity and poor diet) can modify susceptibility to the trait. Health, behavioral, and lifestyle factors [76,77,78,79,80] strongly differ between the general and Roma populations, which also contribute to the difference in life expectancy, but the stronger effect of genetic factors in the case of Roma is supported by our oGRS results. In the lack of follow-up data, the impact of oGRS on longevity could only be measured indirectly by the age of the participant at the time of questionnaire recording. Therefore, a follow-up study would be needed to confirm our results.

## 5. Conclusions

In conclusion, this is the first study to investigate the effect of genetic factors on the development of IR in the Hungarian general and Roma populations. It is concluded that genetic factors influence the development of IR in both populations and that the estimated genetic risk is significantly higher in the Roma population than in the Hungarian general population. It was also found that increased genetic risk in the Roma population is associated with shorter estimated longevity. For both populations studied, effective interventions to prevent IR and T2DM should consider the weight of genetic factors. Furthermore, for the Roma population, high genetic risk might also be associated with early mortality.

## Figures and Tables

**Table 1:** Characteristics of the Roma and Hungarian general populations.

	Roma (*n* = 334)	Hungarian General (*n* = 372)	
	Mean (SD)		*p* value
Age (years)	42.60 (12.18)	44.20 (12.12)	0.114
BMI (kg/m^2^)	27.54 (6.81)	27.21 (5.39)	0.901
Fasting glucose (mmol/L)	5.10 (1.56)	5.27 (1.93)	0.102
Fasting insulin (mU/L)	17.13 (21.02)	15.46 (16.43)	0.891
HOMA—IR	4.45 (7.00)	3.99 (5.54)	0.978
	Prevalence in % (SE)		*p* value
Female	74.6 (2.4)	54.8 (2.6)	0.003
Antihypertensive treatment	30.2 (2.5)	28.5 (2.3)	0.877
Lipid-lowering treatment	12.0 (1.8)	6.7 (1.3)	0.228
Antidiabetic treatment	11.1 (1.7)	5.9 (1.2)	0.245
Elevated fasting glucose (≥7 mmol/L)	9.0 (1.6)	8.1 (1.4)	0.670
Elevated fasting insulin (>20 mU/L)	22.5 (2.3)	20.4 (2.1)	0.499
Elevated HOMA—IR (>3.63)	30.2 (2.5)	29.6 (2.4)	0.846

**Table 2:** The list of SNPs (coded by the most fitting genetic model of inheritance) ordered by the strength of their association (expressed by *p* value from the lowest to the highest) with HOMA—IR in the adjusted (for ethnicity, sex, age, BMI, and treatments) linear regression model.

No.	SNP	Risk Allele	Genetic Model	Beta (95% CI)	*p* Value
1	rs7961581	T	codominant	1.042 (0.406–1.679)	0.001
2	rs1801282	C	dominant	2.084 (0.574–3.594)	0.007
3	rs6822892	G	dominant	0.492 (0.041–0.943)	0.033
4	rs13266634	T	recessive	0.933 (0.070–1.797)	0.034
5	rs4430796	A	codominant	0.574 (0.023–1.125)	0.041
6	rs10010131	A	codominant	0.522 (−0.073–1.117)	0.085
7	rs7903146	T	dominant	0.349 (−0.058–0.756)	0.093
8	rs5219	T	dominant	0.335 (−0.073–0.743)	0.107
9	rs731839	G	recessive	0.502 (−0.139–1.143)	0.124
10	rs459193	A	recessive	0.622 (−0.228–1.471)	0.151
11	rs4402960	T	dominant	0.301 (−0.120–0.722)	0.160
12	rs308971	A	dominant	1.030 (−0.480–2.541)	0.181
13	rs10811661	T	recessive	0.296 (−0.149–0.741)	0.192
14	rs4607103	T	codominant	0.393 (−0.213–0.999)	0.204
15	rs7754840	G	dominant	0.514 (−0.279–1.307)	0.204
16	rs3822072	A	recessive	0.306 (−0.167–0.778)	0.205
17	rs7578597	C	codominant	0.593 (−0.473–1.660)	0.275
18	rs1111875	T	recessive	0.294 (−0.299–0.888)	0.331
19	rs2943645	T	recessive	0.200 (−0.208–0.609)	0.335
20	rs780094	C	codominant	0.240 (−0.320–0.799)	0.401
21	rs4846565	G	recessive	0.163 (−0.246–0.572)	0.433
22	rs10195252	T	recessive	0.155 (−0.258–0.567)	0.462
23	rs10923931	T	dominant	0.188 (−0.319–0.695)	0.467
24	rs8050136	C	dominant	0.164 (−0.360–0.687)	0.540
25	rs4865796	G	recessive	0.188 (−0.546–0.922)	0.615
26	rs864745	C	recessive	0.109 (−0.380–0.597)	0.662
27	rs564398	C	codominant	0.127 (−0.469–0.723)	0.676
28	rs2745353	T	dominant	0.041 (−0.422–0.505)	0.861
29	rs2237892	T	dominant	0.045 (−0.663–0.753)	0.901

**Table 3:** The proportion of people with insulin-resistant (IR) conditions (HOMA—IR > 3.63) by genetic risk categories based on oGRS values in the Roma and Hungarian general populations.

	Low Risk (oGRS: 12 or Less)	Medium Risk (oGRS: 13–15)	High Risk (oGRS: 16 and More)	*p* for Trend
	Prevalence of IR in % (*n*)			
Roma	15.9 (11)	27.4 (48)	46.7 (42)	<0.001
Hungarian general	23.3 (24)	32.5 (55)	31.0 (31)	0.224

Bonferroni corrected *p* value: 0.0033.

## Data Availability

Data available on request due to privacy or ethics.

## Supplementary Materials

The following supporting information can be downloaded at: https://www.mdpi.com/article/10.3390/biomedicines10071703/s1, Figure S1: Linkage disequilibrium map of observed single nucleotide polymorphisms in the Hungarian general (A) and Roma (B) populations, Figure S2: Association of oGRS categories (low-risk group; oGRS: 7–12 was applied as reference one) with risk of insulin resistance (elevated HOMA—IR: > 3.63) as a binary outcome in the Hungarian general and Roma populations. The association was evaluated by using adjusted multinominal logistic regression models. Bonferroni corrected *p* value: 0.0033, Table S1: List of SNPs (from the lowest to the highest *p* value) used for GRS optimization and their effect on the strength of association (expressed as change in *p* value and R square) with HOMA—IR by adjusted (for ethnicity, sex, age, BMI, and treatments) regression models.
