# Supplementary material for: Genetic Susceptibility to Insulin Resistance and Its Association with Estimated Longevity in the Hungarian General and Roma Populations
Source: Biomedicines. 2022 Jul 14;10(7):1703. doi: 10.3390/biomedicines10071703 (PMC9313401; doi:10.3390/biomedicines10071703)
Supplement: Supplementary file 1 [file biomedicines-10-01703-s001.zip › Supplementary Figure S1.pdf]

(A)

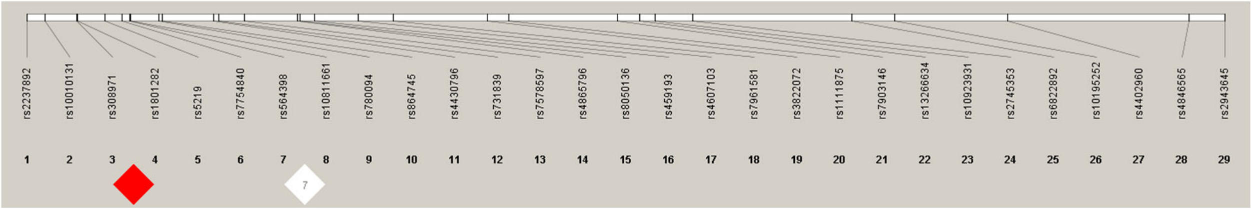

(B)

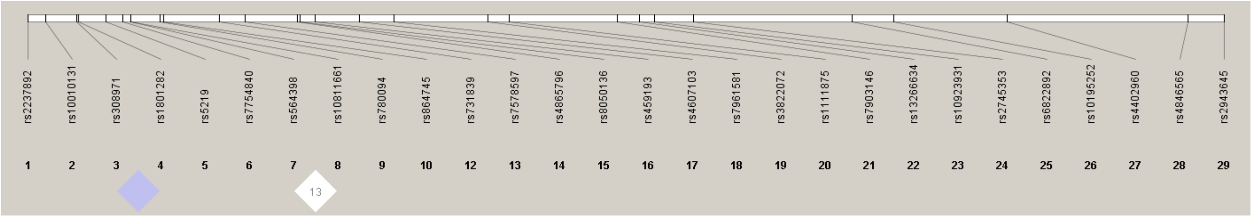

Supplementary Figure S1. Linkage disequilibrium map of observed single nucleotide polymorphisms in the Hungarian general (**A**) and Roma (**B**) populations.
