# Supplementary material for: Genetic Susceptibility to Insulin Resistance and Its Association with Estimated Longevity in the Hungarian General and Roma Populations
Source: Biomedicines. 2022 Jul 14;10(7):1703. doi: 10.3390/biomedicines10071703 (PMC9313401; doi:10.3390/biomedicines10071703)
Supplement: Supplementary file 1 [file biomedicines-10-01703-s001.zip › Supplementary Figure S2.pdf]

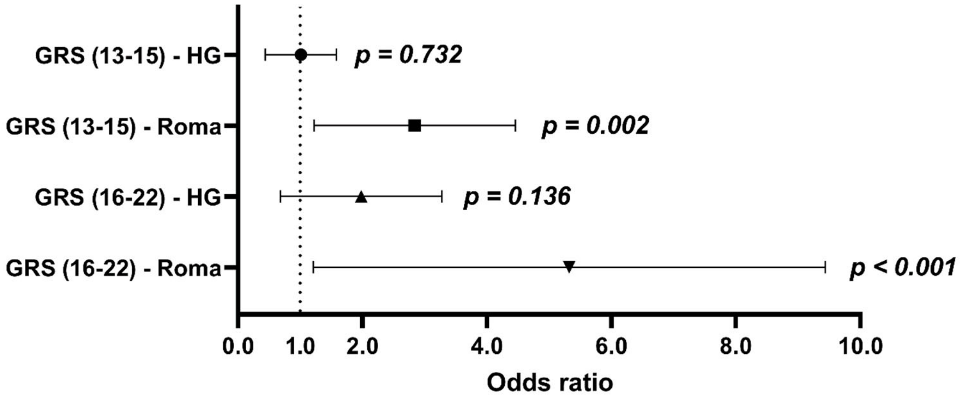

Supplementary Figure S2. Association of oGRS categories (low-risk group; oGRS: 7-12 was applied as reference one) with risk of insulin resistance (elevated HOMA—IR: > 3.63) as a binary outcome in the Hungarian general and Roma populations. The association was evaluated by using adjusted multinominal logistic regression models. Bonferroni corrected *p* value: 0.0033.
