# Supplementary material for: Genetic Susceptibility to Insulin Resistance and Its Association with Estimated Longevity in the Hungarian General and Roma Populations
Source: Biomedicines. 2022 Jul 14;10(7):1703. doi: 10.3390/biomedicines10071703 (PMC9313401; doi:10.3390/biomedicines10071703)
Supplement: Supplementary file 1 [file biomedicines-10-01703-s001.zip › Supplementary Table S1.pdf]

Supplementary Table S1. List of SNPs (from the lowest to the highest *p* value) used for GRS optimization and their effect on the strength of association (expressed as change in *p* value and R square) with HOMA−IR by adjusted (for ethnicity, sex, age, BMI, and treatments) regression models.

**No.**	**SNP**	**Gene**	***p* value**	**R square**	**Delta *p* value**	**Delta R square**	**Included / excluded**
1	rs7961581	*TSPAN8*	1.37E−03	0.2489	——	——	Included
2	rs1801282	*PPARG*	1.02E−04	0.2541	1.27E−03	−5.24E−03	Included
3	rs6822892	*PDGFC*	6.00E−05	0.2552	4.20E−05	−1.07E−03	Included
4	rs13266634	*SLC30A8*	7.00E−06	0.2596	5.30E−05	−4.43E−03	Included
5	rs4430796	*HNF1B*	8.88E−07	0.2638	6.11E−06	−4.16E−03	Included
6	rs10010131	*WFS1*	1.63E−07	0.2672	7.25E−07	−3.46E−03	Included
7	rs7903146	*TCF7L2*	2.05E−07	0.2668	−4.22E−08	4.70E−04	Excluded
8	rs5219	*KCNJ11*	3.66E−07	0.2656	−2.03E−07	1.65E−03	Excluded
9	rs731839	*PEPD*	7.00E−06	0.2595	−6.84E−06	7.77E−03	Excluded
10	rs459193	*C5orf67*	6.89E−08	0.2690	9.42E−08	−1.76E−03	Included
11	rs4402960	*IGF2BP2*	3.50E−08	0.2704	3.39E−08	−1.38E−03	Included
12	rs308971	*SYN2*	1.24E−08	0.2725	2.26E−08	−2.11E−03	Included
13	rs10811661	*CDKN2B−AS1*	1.61E−08	0.2720	−3.68E−09	5.28E−04	Excluded
14	rs4607103	*ADAMTS9−AS2*	8.40E−09	0.2733	4.01E−09	−7.95E−04	Included
15	rs7754840	*CDKAL1*	4.00E−09	0.2748	4.40E−09	−1.51E−03	Included
16	rs3822072	*FAM13A*	4.68E−09	0.2745	−6.80E−10	3.19E−04	Excluded
17	rs7578597	*THADA*	2.23E−09	0.2760	1.77E−09	−1.19E−03	Included
18	rs1111875	*HHEX*	1.91E−09	0.2763	3.20E−10	−3.14E−04	Included
19	rs2943645	*NYAP2*	5.57E−09	0.2741	−3.65E−09	2.17E−03	Excluded
20	rs780094	*GCKR*	2.35E−09	0.2759	−4.36E−10	4.17E−04	Excluded
21	rs4846565	*LYPLAL1−AS1*	3.14E−09	0.2753	−1.22E−09	1.00E−03	Excluded
22	rs10195252	*COBLL1*	1.05E−08	0.2728	−8.59E−09	3.46E−03	Excluded
23	rs10923931	*NOTCH2*	3.93E−09	0.2748	−2.02E−09	1.46E−03	Excluded
24	rs8050136	*FTO*	3.35E−09	0.2751	−1.44E−09	1.14E−03	Excluded
25	rs4865796	*ARL15*	1.88E−09	0.2763	2.79E−11	−3.00E−05	Included
26	rs864745	*JAZF1*	1.13E−09	0.2774	7.59E−10	−1.05E−03	Included
27	rs564398	*CDKN2B−AS1*	2.05E−09	0.2761	−9.28E−10	1.22E−03	Excluded
28	rs2745353	*RSPO3*	1.27E−08	0.2724	−1.15E−08	4.92E−03	Excluded
29	rs2237892	*KCNQ1*	4.37E−09	0.2746	−3.24E−09	2.75E−03	Excluded
